# Comments on: ‘Randomised comparison of a balloon-expandable and self-expandable valve with quantitative assessment of aortic regurgitation using magnetic resonance imaging’

**DOI:** 10.1007/s12471-020-01484-0

**Published:** 2020-08-25

**Authors:** O. Soliman, M. Abdel-Wahab, P. Serruys

**Affiliations:** 1 CORRIB Core Lab and Research Center for Advanced Imaging, National University of Ireland, Galway, Ireland; 2 Department of Cardiology, National University of Ireland, Galway, Ireland; 3 Leipzig Heart Centre, University of Leipzig, Leipzig, Germany

We read with interest the paper by Kooistra et al. [1], who reported on the superiority of a balloon-expandable valve (BEV, SAPIEN 3) over a self-expandable valve (SEV, CoreValve) in terms of aortic regurgitation (AR) and clinical outcome up to 1 year after transcatheter aortic valve implantation (TAVI) in a randomised comparison with quantitative AR assessment using magnetic resonance imaging (MRI).

There are several issues that might have led to bias in the study findings. First, MRI assessment of AR has some limitations, including higher cost, restricted availability, and limited feasibility due to the presence of pacemakers and device implants, compared to other techniques such as quantitative aortography with videodensitometric assessment. The latter has been validated in vitro and in vivo, proven accurate compared to MRI [2] and echocardiography, and is feasible in routine practice in almost every patient [3]. In contrast to MRI, quantitative aortography is readily available as an online tool in the catheterisation laboratory [3]. Comparison of several transcatheter heart valves (THVs) has shown a clear advantage of almost all new-generation THVs compared with an early-generation valve such as the CoreValve [4].

A second issue is the inherent selection bias due to exclusion of 22% of the cases from the final cohort, who were unable to undergo MRI examination in the presence of a pacemaker (PM). Patients with an SEV require PM implantation more often than those with a BEV. Furthermore, patients developing conduction disturbances often have deep implants, which might affect the occurrence and severity of paravalvular leakage (PVL) with both device types. Consequently, a balance between PM need and PVL would be an appropriate objective of a head-to-head device trial. Again, aortography allows assessment of PVL and of depth of implantation, simultaneously. Therefore, aortography could be a better option than MRI for device comparisons in this context.

Furthermore, atrial fibrillation, which was reported in almost one-third of the patients in this paper, reduces the accuracy of flow measurements on MRI. Thus, both phase contrast quantification of AR as well as volumetric quantification could be imprecise.

Most importantly, the choice of an earlier-generation SEV that is known to be inferior to the novel-generation BEV in terms of PVL and PM need represents an a priori advantageous bias toward the newer-generation device. A recent publication by Modolo et al. on 2258 patients with seven different devices, as well as other earlier publications, show the highest incidence of PVL after early-generation SEV [4]. Thus, it would be beneficial to compare contemporary device generations such as the Evolut series versus the SAPIEN 3 series. Unfortunately, the authors stopped enrolment in this study upon availability of the CoreValve Evolut series, an SEV with an improved design and better sealing properties against PVL [4].

Despite our critical comments, we must commend the authors for exploring ways to promote objective device head-to-head comparisons in the field of TAVI and for furthering the discussion about the effects of AR on outcome. However, we maintain our call for the use of quantitative aortography, a fully validated technique for AR assessment, as it essentially eliminates unnecessary costs and overcomes the limited availability and/or feasibility of MRI in daily patient care.
